# Transcriptomic profiling during normothermic machine perfusion of human kidneys reveals a pro-inflammatory cellular landscape and gene expression signature associated with severe ischemia-reperfusion injury and delayed graft function

**DOI:** 10.3389/fimmu.2025.1679251

**Published:** 2025-10-22

**Authors:** Harry V. M. Spiers, Sarah A. Hosgood, Miguel Larraz, Lukas J. K. Stadler, Ying Zhai, Haeun Moon, Serena MacMillan, Anna Paterson, Michael L. Nicholson, Irina Mohorianu, Vasilis Kosmoliaptsis

**Affiliations:** ^1^ Department of Transplantation, Addenbrooke’s Hospital, Cambridge, United Kingdom; ^2^ Department of Surgery, University of Cambridge, Cambridge, United Kingdom; ^3^ NIHR Blood and Transplant Research Unit in Organ Donation and Transplantation at the University of Cambridge, Cambridge, United Kingdom; ^4^ Department of Histopathology, Addenbrooke’s Hospital, Cambridge, United Kingdom; ^5^ Cambridge Stem Cell Institute, University of Cambridge, Cambridge, United Kingdom; ^6^ School of Electronics and Computer Science, Faculty of Engineering and Physical Sciences, University of Southampton, Southampton, United Kingdom; ^7^ Clinical and Experimental Sciences, Faculty of Medicine, University of Southampton, Southampton, United Kingdom

**Keywords:** ischemia reperfusion-injury, delayed graft function, normothermic machine perfusion, RNAseq, gene set enrichment analysis, innate immunity

## Abstract

**Background:**

Assessment and treatment of severe ischemia-reperfusion-injury (IRI) remains an unmet challenge in kidney transplantation. Normothermic machine perfusion (NMP) recapitulates IRI *ex situ*, but there is limited understanding of the transcriptional pathways, and the associated cellular landscape, driving IRI during NMP and determining its severity. Such knowledge is essential for therapeutic targeting and organ resuscitation during machine perfusion.

**Methods:**

Using tissue obtained at the time of NMP from kidneys subsequently transplanted as part of a randomized controlled trial, we undertook in-depth transcriptomic analyses comparing kidneys suffering severe IRI, (manifesting clinically as the development of delayed graft function (DGF)), to kidneys with mild IRI (defined by immediate graft function, IGF) post-transplantation.

**Results:**

We validated upregulation of previously described pro-inflammatory and immune transcriptomic pathways, including *TNFa via NFkB signaling, Allograft Rejection* and *Inflammatory Response*. Going further, we identified innate immune system driven processes at the core of the transcriptional signature in kidneys suffering severe IRI, such as recruitment and migration of myeloid leucocytes, macrophage activation, phagocytosis and inflammasome activation. Deconvolution using single-cell-RNAseq data showed kidneys with severe IRI and post-transplant DGF were enriched for pro-inflammatory mononuclear phagocytes, myofibroblasts and fibroblasts, but depleted of tubuloepithelial, cell signatures. These transcriptional findings were recapitulated in tissue biopsies obtained during NMP from an external cohort comparing kidneys with high acute tubular injury and severe IRI to kidneys with low acute tubular injury and mild IRI; these kidneys were histologically similar to the DGF/IGF kidneys, respectively.

**Discussion:**

Together, our study characterizes the transcriptional signature of severe IRI during NMP, suggesting the role of pro-inflammatory innate/pro-fibrotic cells in this process. We describe a transcriptomic signature that may support future prospective therapeutic trials as a potential efficacy endpoint, and highlight potential cellular targets for therapeutic intervention during NMP in an era of precision medicine.

## Introduction

Kidney transplantation (KT) represents the optimal treatment for patients with end stage renal disease. It offers superior morbidity (1), survival (2), quality of life (1) and health economic (3) outcomes compared to dialysis. However, organ shortage necessitates increased use of marginal kidneys from extended criteria and donation after circulatory death (DCD) donors. These suffer an enhanced ischemia-reperfusion injury (IRI), clinically manifesting as delayed graft function (DGF) (4, 5). DGF in turn is associated with poor long-term outcomes, including inferior long-term graft function, increased rates of rejection and graft loss (4, 5).

Normothermic machine perfusion (NMP) can be used to assess kidneys prior to transplant (6), and its feasibility and safety in clinical kidney transplantation has been established at the randomized controlled trial (RCT) level (7). NMP recapitulates IRI *ex situ* providing a unique isolated organ platform for the study of IRI, and its experimental modulation. In previous analysis of tissue obtained at the time of NMP during the aforementioned RCT, kidneys that developed DGF had a different transcriptional profile compared to kidneys with immediate graft function (IGF) (8). Specifically, during NMP, kidneys that developed DGF requiring dialysis in only the first 24 hours post-transplant shared transcriptomic profiles with IGF kidneys, whilst those requiring dialysis beyond 24hrs had a distinct inflammatory profile (8).

All kidneys suffer an inevitable IRI during NMP, the degree of which correlates with pre-existing kidney injury and with graft function post-transplant. In an era of therapeutic intervention during NMP, understanding the mechanisms and cellular landscape underpinning IRI may aid development of novel and cell-specific therapeutics. This could be an important step towards addressing an unmet need, namely therapeutic targeting of the inherent IRI in kidney transplantation. In this study, we used unbiased transcriptomic analyses of human kidney biopsies taken during NMP, to characterize transcriptomic signatures related to the severity of IRI, and correlated these to post-transplant clinical outcomes and to the degree of kidney injury on histology. Furthermore, we used publicly available single-cell-RNAseq data to deconvolute tissue transcriptomic signatures and outline the cellular landscape of severe IRI in kidneys undergoing NMP. This work investigates the transcriptomic landscape of kidney IRI during NMP and may be used in future studies of IRI and its therapeutic manipulation.

## Methods

### Experimental design

To explore the transcriptomic signature of severe IRI during NMP, we used samples from a biobank of DCD kidneys perfused as part of the only randomized controlled trial of kidney NMP (7). We selected kidneys expected to have low IRI, given their immediate graft function after transplantation (primary function or hemodialysis for less than 24 hours post-transplant, n=10), and transcriptomically compared them to those with severe IRI, clinically manifested as delayed graft function (hemodialysis beyond 24 hours, n=10). In the absence of an external clinical cohort of kidneys that underwent NMP prior to transplantation, we then sought to validate findings in an external research cohort of NMP kidneys (initially accepted but subsequently declined for transplantation). This included 7 paired kidneys previously randomized in a 1:1 fashion to undergo perfusion with either red blood cell (RBC) based perfusate, or an acellular perfusate; the full analysis of these kidneys is beyond the scope of this study, but important details are provided below. Using the degree of acute tubular injury as a histological surrogate of IRI/DGF (9–12), the kidneys were assessed and grouped into low (n=6) and high (n=7) acute tubular injury cohorts, which were histologically similar to IGF and DGF kidneys respectively (discussed below), comprising the validation cohort.

### Clinical NMP kidney trial cohort

To further explore the tissue transcriptome of IRI during NMP, samples from a biobank procured as part of the aforementioned RCT (7), were utilized (**Supplementary Figure S1A**). For bulk RNAseq, tissue biopsies taken at the end of NMP (1 hour of NMP) were selected for 10 IGF and 10 DGF kidneys. Following total RNA extraction and quality assessment, nine samples (IGF n=6, DGF n=3) yielded suitable RIN values (≥6) and were sequenced on an Illumina NovaSeq6000 platform. Following post-sequencing quality control, 7 samples (IGF n=4, DGF n=3) were suitable for analysis. One further DGF sample was excluded based on poor graft function explained by severe acute cellular rejection on day 7 post-transplant. The final cohort included for bulk RNAseq analysis comprised 6 samples (n=4 IGF, n=2 DGF), summarized in **Supplementary Figure S1A**.

#### NMP kidney trial protocol and definitions

Ethical approval for the previously completed trial (7) and collection of biological samples was granted by the East of England Cambridge Central Research Ethics Committee (15/EE/0356). Approval was specifically granted for a multicenter UK based open label randomized controlled trial of NMP in DCD kidney transplantation (7). Trial Registration Number: ISRCTN15821205. Recruitment was open to eligible patients receiving a controlled DCD kidney transplant (Maastricht Categories III & IV). All kidneys were procured following a National Organ Procurement Service protocol. Patients were randomized in a 1:1 fashion to receive a kidney either statically cold stored (SCS, n=200) or SCS followed by one hour of NMP (n=200). In the current study, only a subcohort of the NMP arm was available within the remaining biobank. Delayed graft function was defined as the need for hemodialysis within the first 7 days post-transplant. However, previous work (8) showed no transcriptomic difference between kidneys experiencing immediate graft function (IGF) and those labelled as DGF due to hemodialysis within the first 24hrs (most commonly due to hyperkalemia). Therefore, this study defined IGF as either primary function or hemodialysis for <24hrs post-transplant, and delayed graft function (DGF) as dialysis required beyond 24 hours post-transplant.

#### NMP kidney trial perfusion protocol

The full perfusion protocol for RBC based NMP used in the RCT was published by Hosgood et al (7). In brief, after a period of SCS on ice at 4°C, the kidneys were weighed and prepared for perfusion. Renal artery and ureter were cannulated, and kidneys flushed with 1L of cold Ringer’s solution to remove preservation solution. Kidneys were then perfused using an adapted pediatric cardiac bypass system (Medtronic, Bioconsole 560). The circuit was primed with 300ml Ringer’s solution (Baxter Healthcare, Thetford, UK), 15ml Mannitol 10% (Baxter Healthcare), 27 ml sodium bicarbonate 8.4% (Fresenius Kabi, Runcorn, UK), 3000iu heparin (LEO Pharma A/S, Ballerup, Denmark) and 6.6mg Dexamethasone (Hameln Pharmaceuticals, Hamelin, Germany). A unit of ABO compatible packed red cells was then added. The perfusion solution was oxygenated (95% oxygen/5% CO_2_) at a flow rate of 0.1L/min and warmed to 35.5 - 36.5°C. Perfusion was undertaken continuously through the renal artery at a mean arterial pressure of 85mmHg and pump speed of 1450RPM. Nutrients (Synthamin 17 10%, Baxter Healthcare, Thetford, UK) and 15mls of sodium bicarbonate 8.4% (B Braun, Melsungen, Germany) were added. Insulin (100IU; Actrapid, Novo Nordisk, London, UK) was infused at a rate of 20ml/h, in addition to glucose 5% (Baxter Healthcare), at a rate of 5ml/h and Ringer’s solution was used to replace urine output (ml for ml).

### Research kidney NMP validation cohort

An external cohort of available machine perfused kidneys were used as a validation cohort. Seven pairs of kidneys procured for transplant but subsequently declined and offered for research were randomly assigned to perfusion with either a red blood cell (RBC) based perfusate, or an acellular based perfusate. All kidneys were procured following a National Organ Procurement Service protocol and transported under SCS. Ethical approval for the study was granted by the national ethics committee in the UK REC (22/WA/0167).

#### Machine perfusion within the validation cohort

Following SCS kidneys were weighed and prepared for perfusion in the same fashion as those from the kidney NMP trial above. The same perfusion system as above was used for both RBC and acellular perfused kidneys, with perfusion undertaken at 32°C for six hours. RBC perfused kidneys received perfusate as per the NMP RCT. Acellular perfused kidneys were perfused with a Human serum albumin (5%, 250ml) and Ringer’s solution (219ml) based perfusate, supplemented with 6.6mg dexamethasone (Hameln Pharmaceuticals, Hamelin, Germany), 5ml calcium gluconate 10% and 15ml sodium bicarbonate 8.4% (Fresenius Kabi, Runcorn, UK), 250mg meropenem and 2.5mg verapamil. During perfusion Synthamin 17 10% (Baxter Healthcare, Thetford, UK) supplemented with 15mls of sodium bicarbonate 8.4% (B Braun, Melsungen, Germany), 5ml multivitamins, and 1ml insulin (100IU; Actrapid, Novo Nordisk, London, UK) was infused at 10ml/hr. Glucose 5% (Baxter Healthcare), at a rate of 3ml/h, and Glyceryl trinitrate (10ml of 50mg/ml in 90ml Saline) at 10ml/hr for the first hour then 5ml/hr thereafter, were also infused. Ringer’s solution was used to replace urine output (ml for ml).

#### Sample processing for bulk RNAseq analysis

In the validation cohort, RNA was extracted from tissue biopsies taken from seven kidney pairs at the end of SCS prior to commencement of NMP and at the end of 6 hours of NMP. All samples had a RIN value of ≥6 and proceeded to sequencing. Following QC, a total of 25 samples were suitable for downstream analysis (pre-NMP n=12, 6hrs NMP n=13; **Supplementary Figure S1B**).

### Histological assessment

Histological slides of all IGF (n=4) and DGF (n=2) kidneys from the NMP trial cohort included in the bulk RNAseq analysis, were reviewed by a blinded specialist transplant pathologist (AP). We hypothesized that kidneys with IGF were more likely to have lower distribution and severity of acute tubular injury (ATI), and DGF kidneys more likely to have wider distribution and severity of ATI; blinded review of histology confirmed this, with IGF kidneys better preserved in terms of ATI than DGF kidneys. Blinded review of histology at the end of 6 hours of NMP in the validation cohort saw each kidney graded by distribution and severity of ATI, and the cohort ranked from lowest to highest ATI. The cohort was divided at the midpoint into low (n=6) and high (n=7) ATI cohorts. This resulted in a validation cohort with low ATI, histologically similar to IGF kidneys, and high ATI, histologically similar to DGF kidneys.

### Sample storage and RNA extraction

Tissue biopsies were stored in RNAlater solution (Invitrogen RNAlater Solution) prior to transfer to -80°C until processing. Samples were thawed on ice, removed from storage solution (if applicable), weighed, and then macerated on ice using a sterile scalpel. Macerated tissue was transferred to 700uL QIAzol Lysis Reagent (Qiagen, Hilden, Germany) and homogenized using a sterile pestle followed by further homogenization via 10x passages through a 20g sterile needle. 140uL of chloroform was added and sample shaken for 15 seconds by hand before being centrifuged at 12,000g for 15 minutes at 4°C. The clear upper supernatant containing RNA was taken forward and 1.5x the volume of 100% ethanol was added. This was then loaded onto Qiagen RNeasy Midi columns and total RNA extracted according to manufacturer guidance. RNA was eluted in 50uL of RNAse free water. DNA was removed post-RNA extraction using a TURBO DNA-free™ kit (Invitrogen™). Quality of RNA was assessed using a nanodrop spectrophotometer and an RNA nano Bioanalyzer kit (Agilent Technologies, Palo Alto, CA, USA) using a Bioanalyzer 2100 (Agilent Technologies, Palo Alto, CA, USA). Samples with RIN >6 were sent for sequencing.

### RNA sequencing

RNA was shipped on dry ice to Genewiz Azenta (Germany) for RNA sequencing. Once received, RNA samples were quantified using Qubit 4.0 Fluorometer (Life Technologies, Carlsbad, CA, USA) and RNA integrity was checked with RNA Kit on Agilent 5300 Fragment Analyzer (Agilent Technologies, Palo Alto, CA, USA). rRNA depletion was performed using QIAGEN FastSelect rRNA HMR Kit (Qiagen, Hilden, Germany). RNA sequencing library preparation was performed with NEBNext Ultra II RNA Library Preparation Kit for Illumina following the manufacturer’s recommendations (NEB, Ipswich, MA, USA). Briefly, enriched RNAs were fragmented for 15 minutes at 94°C. First strand and second strand cDNA were subsequently synthesized. cDNA fragments were end repaired, adenylated at 3’ends, and universal adapters were ligated to cDNA fragments, followed by index addition and library enrichment with limited cycle PCR. Sequencing libraries were validated using the Agilent Tapestation 4200 (Agilent Technologies, Palo Alto, CA, USA), and quantified using Qubit 2.0 Fluorometer (ThermoFisher Scientific, Waltham, MA, USA) as well as by quantitative PCR (KAPA Biosystems, Wilmington, MA, USA).

The sequencing libraries were multiplexed and loaded on the flowcell on the Illumina NovaSeq 6000 instrument according to manufacturer’s instructions. The samples were sequenced using a 2x150 Pair-End (PE) configuration v1.5. Image analysis and base calling were conducted by the NovaSeq Control Software v1.7 on the NovaSeq instrument. Raw sequence data (.bcl files) generated from Illumina NovaSeq was converted into fastq files and de-multiplexed using Illumina bcl2fastq program version 2.20. One mismatch was allowed for barcode matching.

### Transcriptomic analysis

The raw RNAseq samples were subjected to preprocessing comprising subsampling without replacement (13) to 75M reads and trimming forward and reverse reads to 100 nucleotides (nts), which reduced adapter contamination to within accepted levels (<5% across samples). The proportion of retained biological signal, before and after each processing step, was assessed using fastQC (14), summarized using multiQC (15). Poor quality samples, from a technical QC perspective, included two IGF samples with significantly higher overall GC% *(p* = 0.025, under a standard t-test), calculated across the retained reads, as well as low numbers of unique reads indicating degradation; the mapping was performed using STAR (v2.7.4a) (16), with default parameters against vHg38 of the *H sapiens* genome. MultiQC was used to aggregate the quality reports from the fastQC, alignment and gene quantification, with ~85% reads mapping uniquely across samples. The quantification of genes, based on the *H Sapiens* gtf annotation, was performed using featureCounts (17). The two samples with poor RNA QC retained their poor assessment throughout mapping and quantification and were excluded from downstream analyses. An interactive bulkAnalyseR app (18) was subsequently generated on the retained samples; noise correction at expression-matrix level was performed using *noisyR* (19); the normalization of gene expression was performed using quantile normalization (20).

Differential expression analysis was performed using *DESeq2 (* 21) and *edgeR (* 22). A log2(FC) threshold cut off of 0.75 and adjusted p-value 0.05, using Benjamini-Hochberg multiple testing correction, were used to determine DE mRNA transcripts for subsequent analyses. For gene set enrichment analysis (GSEA), all differentially expressed genes (log2(FC) threshold 0) were ranked by the inverse of the p value with the sign of the log fold-change, then ran against the Hallmark’s database within MSigDB (23), using the GSEA tool from the Broad Institute (24) with the pre-ranked option, and the following parameters: *No_collapse, classic, default settings*. Over-representation analysis was performed using *gProfiler2 (* 25), with ranked genes ran against the Gene Ontology, Kyoto Encyclopedia of Genes and Genomes (KEGG) (26) and Reactome Pathway (REAC) (27) databases, using all genes expressed in the deriving samples as background set. Single sample GSEA (*ssGSEA*) was carried out using normalized counts from quantile normalization using the GenePattern platform (28).

### STRING analysis

Protein coding genes, identified based on their Ensembl IDs (ENSG) were converted to protein names and STRING (29) analysis undertaken. Two proteins were considered connected if they were part of the same physical subnetwork and had a mean interaction score of >0.7. Unconnected proteins were removed from the network and force direction graph plotted. The resultant network was subdivided using MCL clustering based on stochastic flow, and clusters annotated using selected GO terms associated with each cluster.

### Statistical analysis of clinical data

Continuous clinical data were presented as median (interquartile range, IQR), with comparisons between groups performed using the Mann-Whitney U test. For categorical data, comparisons between groups were performed using the Fisher’s exact or Chi-Square tests as appropriate. A two-sided p-value <0.05 was considered statistically significant. These analyses were performed using the *finalfit* package in R studio (Version 4.4.3 – *Trophy Case*).

## Results

The study aim was to investigate the transcriptomic signatures and cellular landscape underpinning IRI during human kidney NMP, using post-transplant kidney graft function/dysfunction as a clinical correlate of IRI severity. We aimed to outline the mechanisms of IRI at play during NMP rather than delivering biomarkers of graft function post-transplant, which this study is not powered for.

Deceased-donor demographics and relevant clinical course post-transplant for the entire cohort (n=20) are presented in **Supplementary Table 1**. Compared to donors of kidneys with IGF, DGF kidney donors were predominantly male (30% vs 70%, p=0.179), had higher BMI (25.9 vs 30.2,p=0.104) and longer cold ischemic times (796.5 vs 925.5,p=0.226). Recipients with DGF compared to IGF had lower eGFR at 6 months (47.5mls/min vs 36.0 mls/min, p=0.558), and 12 months (50.5mls/min vs 42.0 mls/min, p=0.773). Full analysis is provided in **Table 1**.

**Table 1:** Demographics of kidney donors and post-transplant outcomes.

Whole clinical cohort		*IGF*	*DGF*	*P value*	RNAseq analysis cohort	*IGF*	*DGF*	*P value*
*Total n (%)*	-	10 (50.0)	10 (50.0)	-		4 (66.7)	2 (33.3)	-
*Donor age (yrs)*	Median (IQR)	57.0 (52.0 to 61.8)	58.5 (44.5 to 64.5)	0.910		49.5 (43.0 to 52.0)	67.5 (63.8 to 71.2)	0.064
*Gender*	Female	7 (70.0)	3 (30.0)	0.179		3 (75.0)	0 (0.0)	0.400
	Male	3 (30.0)	7 (70.0)			1 (25.0)	2 (100.0)	
*BMI (kg/m2)*	Median (IQR)	25.9 (23.7 to 29.4)	30.2 (28.6 to 33.4)	0.104		28.6 (25.8 to 33.6)	31.0 (28.9 to 33.0)	1.000
*Donor Cause of Death*	Hypoxic brain damage	5 (50.0)	3 (30.0)	0.867		2 (50.0)	0 (0.0)	0.733
	Intracranial thrombosis	1 (10.0)	1 (10.0)			0 (0.0)	1 (50.0)	
	Intracranial haemorrhage	3 (30.0)	3 (30.0)			1 (25.0)	1 (50.0)	
	Cardiac arrest	1 (10.0)	1 (10.0)			1 (25.0)	0 (0.0)	
	Other	0 (0.0)	2 (20.0)			0 (0.0)	0 (0.0)	
*Pre-retrieval Creatinine (mg/dl)*	Median (IQR)	70.0 (53.0 to 89.8)	73.0 (64.8 to 110.0)	0.650		66.0 (55.0 to 112.5)	96.0 (84.5 to 107.5)	0.643
*Warm Ischaemia Time (mins)*	Median (IQR)	13.0 (10.0 to 14.0)	14.0 (12.0 to 15.0)	0.328		10.0 (9.5 to 12.0)	13.0 (12.5 to 13.5)	0.374
*Cold Ischaemia Time (mins)*	Median (IQR)	796.5 (588.5 to 905.8)	925.5 (761.8 to 1067.0)	0.226		898.5 (821.8 to 979.0)	1132.0 (1110.5 to 1153.5)	0.355
*Anastomosis Time (mins)*	Median (IQR)	39.5 (27.2 to 48.8)	40.5 (32.0 to 50.8)	0.596		39.5 (33.2 to 45.2)	44.5 (41.2 to 47.8)	0.355
*6 month eGFR (mls/min)*	Median (IQR)	47.5 (41.5 to 49.0)	36.0 (29.0 to 56.0)	0.558		49.0 (48.2 to 49.5)	29.0 (26.0 to 32.0)	0.060
*12 month eGFR (mls/min)*	Median (IQR)	50.5 (48.5 to 54.8)	42.0 (39.0 to 62.0)	0.773		54.0 (53.0 to 55.5)	32.5 (29.2 to 35.8)	0.083

Table outlining comparison of IGF and DGF donors and selected recipient outcomes for the whole clinical cohort, and for kidneys specifically included in the RNAseq analysis. BMI, body mass index; DGF, delayed graft function; IGF, immediate graft function; IQR, inter-quartile range.

**Figure 1:**
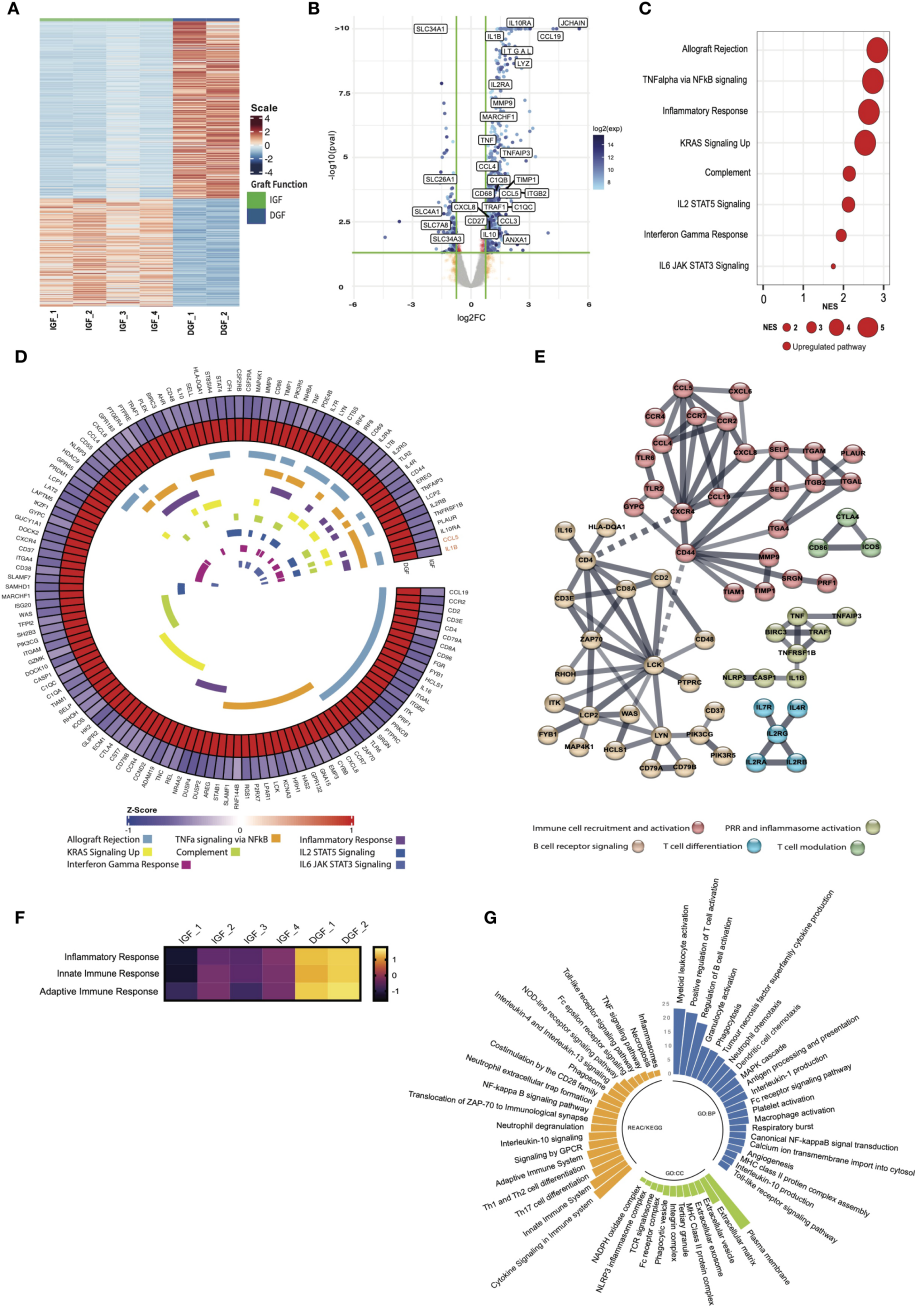
Bulk RNA transcriptional analysis reveals immune and inflammatory pathway activation drives the core signature of severe IRI/delayed graft function. **(A)** Heatmap demonstrating differentially expressed genes between IGF and DGF kidneys (**Supplementary Table 2**); scale represents Z-scored expression. **(B)** Volcano plot of genes differentially expressed in DGF kidneys, annotated with upregulated pro-inflammatory and immune related genes, and downregulated solute transporter genes. Exp; expression. **(C)** Gene Set Enrichment Analysis (GSEA) on differentially expressed genes (logFC1.5,p value<0.05) against the MSigDB Hallmarks dataset (23), in DGF kidneys reveals immune and inflammatory pathway activation driving severe IRI (**Supplementary Table 3**). Only significant pathways are plotted (FDR <0.05). Size of bubble corresponds to degree of enrichment. NES, normalized enrichment score; FDR, false discovery rate. **(D)** Circular heatmap of leading edge genes from enriched pathways in **(C)** internal colors represent pathways each specific gene is annotated to; genes highlighted in orange (*CCL5, IL1B*) show greatest cross-pathway annotation (five pathways; **Supplementary Table 4**). **(E)** STRING analysis of leading edge genes from C; solid line represents interaction within protein cluster, dashed line represents interaction between clusters. **(F)** Heatmap of scaled enrichment of pathways from single sample GSEA. **(G)** Bar plot of pathways over-represented in DGF kidneys using significantly differentially expressed genes (**Supplementary Table 5**).

Following RNA extraction, quality assessment and post-sequencing quality control (see methods), 7 samples were suitable for further transcriptomic analysis (n=4 IGF, n=3 DGF, see methods, **Supplementary Figure S1A** and **Supplementary Figures S2A–D**). Initial transcriptomic comparison of IGF and DGF kidneys revealed similar gene expression between groups (**Supplementary Figure S2E**), confirmed on principal component analysis (PCA, **Supplementary Figure S2F**). This similarity could be attributed to the effect of NMP following cold storage, leading to common transcriptional changes consistent with reperfusion. Examination of differentially expressed genes (DEGs) highlighted one DGF kidney as transcriptionally dissimilar to the others in the group, particularly in terms of upregulated genes, better aligning to the transcriptional profile of IGF kidneys (**Supplementary Figure S2G**). Investigation of clinical data revealed the etiology of DGF to be acute T-cell mediated rejection, as confirmed at biopsy on day 7 post-transplant, which showed absence of acute tubular ischemia or necrosis. Notably, graft function significantly improved following treatment with methylprednisolone and immunosuppression modulation. Given graft dysfunction was likely due to a memory immune response, rather than true DGF secondary to IRI driven acute tubular injury (ATI), this kidney was excluded from the DGF cohort, ensuring those included in the analysis had DGF as a consequence of ATI and severe IRI. In the final cohort included for analysis, compared to IGF kidney donors, the DGF kidney donors were older (49.5 vs 63.8 years, p=0.064), all male (25% vs 100%, p=0.4), had longer CIT (898.5mins vs 1132.0mins,p=0.355), and had lower eGFR at 6 months (49mls/min vs 29mls/min, p=0.06) and 12 months (54mls/min vs 32.5mls/min, p=0.083). The final cohort showed differences between IGF and DGF on PCA (**Supplementary Figure S2I**), with intra-group similarity of DEGs (**Figure 1A**). Recipients of the kidneys developing DGF had at least 7 days of hemodialysis and acute tubular necrosis confirmed on biopsy at day 7 post-transplant.

### Immune and inflammatory pathway activation drives the core signature of DGF

We found 841 DEGs in DGF compared to IGF kidneys, including 674 upregulated and 167 downregulated genes (**Figure 1B**, **Supplementary Table 2**). Upregulated genes comprised those encoding pro-inflammatory cytokines (*TNF, IL1B, CXCL8/IL8)*, immune cell recruiting chemokines (*CCL3, CCL4, CCL5*), integrin-mediated leucocyte adhesion molecules (*ITGB2, ITGAL*), and extracellular matrix remodeling proteins (*MMP9, TIMP1*). Genes associated with monocytic lineage (*CD68, LYZ*), T cells (*IL2RA, MARCHF1)* and B cells (*JCHAIN, CCL19*) were also upregulated, as were complement associated genes (*C1QB, C1QC*). Interestingly, genes associated with anti-inflammatory/immune regulatory responses (*IL10, IL10R, TNFAIP3*) were also upregulated compared to IGF kidneys. Downregulated genes included those associated with tubular cell function, specifically solute transport (*SLC4A1, SLC7A8, SLC34A3*).

Gene set enrichment analysis (GSEA) using all DEGs in DGF kidneys recapitulated the enrichment of pro-inflammatory pathways previously shown in kidneys from the same RCT (8), including *TNFa via NFkB signaling* and *Inflammatory response*, whilst confirming depletion of *Oxidative phosphorylation* (**Supplementary Figure S3**). These findings recapitulate the global transcriptomic signature of IRI previously shown in DGF kidneys during NMP (8). Next, we performed GSEA using only protein coding genes that were significantly differentially expressed in DGF kidneys, to establish a core transcriptional signature of severe IRI (**Figure 1C**). This demonstrated persistent enrichment of eight immune and inflammatory pathways, including *Allograft rejectio*n, *TNF alpha signaling via NFkB* and *Inflammatory response* (**Figure 1C**). Leading-edge analysis of these eight pathways driving severe IRI identified 133 unique driver genes (**Supplementary Table 4**). When exploring overlapping gene expression between pathways (**Figure 1D**), only two genes were part of 5 or more pathways: *CCL5*, an immune cell recruitment chemokine, specifically for CCR5-, CXCR3- and CXCR5-expressing lymphocyte subsets, and *IL1B*, a potent pro-inflammatory cytokine secreted by pro-inflammatory (M1) macrophages. STRING analysis of leading-edge genes (**Figure 1E**) showed five major nodes: immune cell recruitment and activation, pattern recognition receptor (PRR) and inflammasome activation, B cell receptor signaling, T cell differentiation, and T cell modulation. Taken together, these results highlight a pro-inflammatory immune mediated injury underpinning severe IRI during NMP in kidneys going on to develop DGF, with both innate and adaptive immune response components. This signature is present after just one hour of NMP and may reflect a significantly up/dysregulated innate immune process, supported by leading-edge genes that translate to cytokines/chemokines secreted by cells of myeloid lineage, and activation of TNFalpha and NFkB signaling pathways.

### Kidneys with severe IRI show enrichment for innate immune processes driven by NFkB signaling during NMP

Having established a core transcriptomic signature of severe IRI in kidneys during NMP, we sought to better characterize the underpinning mechanisms, employing over-representation analysis (ORA) querying the Gene Ontology, KEGG, and Reactome databases, to investigate the functional role of genes and related pathways driving the transcriptomic signature (**Supplementary Table 5**). Severe IRI kidneys were enriched for inflammatory response and both innate and adaptive immune responses, with many of the enriched pathways linked to these higher order terms in the gene ontology database (**Figure 1F**). Kidneys with severe IRI displayed enrichment of terms associated with cytokine production and leucocyte chemotaxis, corroborating STRING analysis. These organs were also enriched for recruitment and migration of myeloid leucocytes, macrophage activation and phagocytosis, further evidence of innate immune activation in IRI (**Figure 1G**). Furthermore, we found enrichment of pro-inflammatory signaling, specifically through *MAPK cascade*, *Canonical NF-kappaB signal transduction*, and *Toll-like receptor signaling pathway*. Enriched cellular components suggested cell interaction through integrins and remodeling of the extracellular matrix (**Figure 1G**). Innate immune related components such as *phagocytic vesicle* and *NLRP3 inflammasome* were also enriched. Genes upregulated in severe IRI were also annotated to extracellular vesicles (EVs), promising biomarkers and potential therapeutic/drug delivery systems in transplantation (30). Querying KEGG and Reactome pathway databases further highlighted the role of NFkB signaling in severe kidney IRI during NMP (**Figure 1G**), in addition to enrichment of PRR activation, a common signaling event initiating NFkB pathway activation. There was also enrichment in the downstream consequences of NFkB activation, including cytokine signaling, *Th17 cell differentiation* and *inflammasome activation*. Importantly, although most pathways enriched in severe IRI are pro-inflammatory, we identified enrichment of *Interleukin-10* and *Interleukin-4 and interleukin-13 signaling*, both involved in resolution of the immune/inflammatory response, promoting anti-inflammatory/pro-repair ‘M2’ macrophage polarization (31). Taken together, our results reveal a strong innate immune signature underpinning severe IRI during NMP in kidneys developing DGF post-transplant, including innate-adaptive immune crosstalk and propagation of inflammation.

### Mononuclear phagocytes are enriched in kidneys during NMP that develop DGF

**Figure 2:**
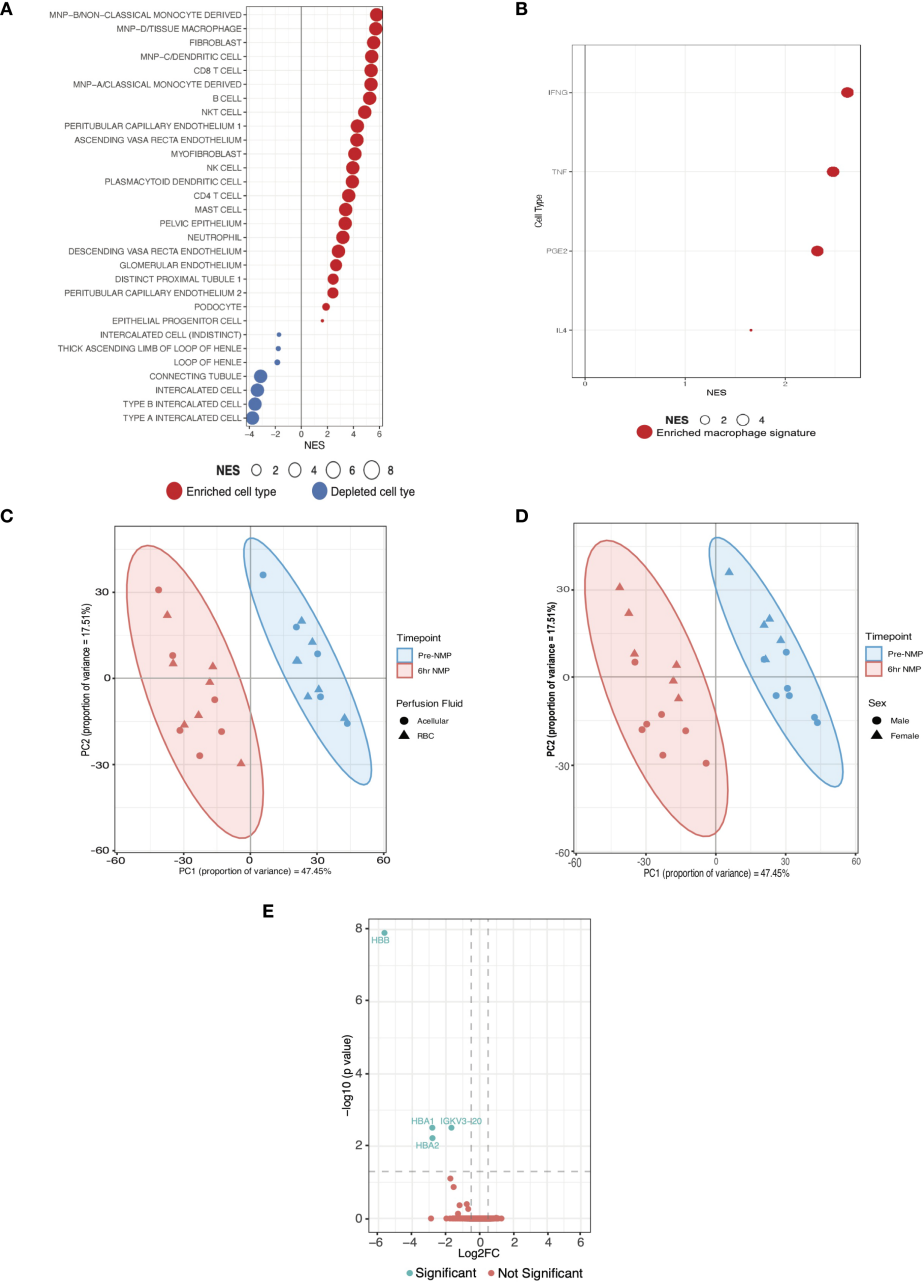
Cell expression signatures enriched in kidneys with severe IRI that develop delayed graft function and establishment of a validation cohort. **(A)** GSEA of differentially expressed genes in DGF using curated scRNAseq signatures of kidney cell types from a spatio-temporally resolved human kidney atlas (**Supplementary Tables 6, 8**) (32). Only significant cell types are plotted (FDR <0.05). Size of bubble corresponds to degree of enrichment. NES, normalized enrichment score; FDR, false discovery rate. **(B)** GSEA of the same genes as A using cytokine-specific macrophage polarization signatures (**Supplementary Table 7**); only significant cell types are plotted (FDR <0.05). **(C)** Principal component analysis (PCA) of top 500 genes by expression on perfusion timepoints (i.e. pre-NMP and at the end of 6hrs-NMP), annotated for perfusate, in the validation cohort. **(D)** PCA of top 500 genes by expression on perfusion timepoints (i.e. pre-NMP and at the end of 6hrs-NMP), annotated for donor sex, in the validation cohort. **(E)** Volcano plot of differentially expressed genes between RBC and acellular perfused kidneys in the validation cohort.

Having established NFkB signaling as a key upregulated pathway in kidneys suffering more severe IRI and developing DGF, and an associated enrichment of innate immune system pathways, we sought to infer the cellular landscape underpinning severe IRI during NMP. We used curated cell-type signatures from a spatiotemporally resolved kidney atlas (32) (**Supplementary Table 6**) to deconvolute our bulk RNAseq data. Mononuclear phagocyte (MNP) signatures were found to be enriched in severe IRI kidneys (**Figure 2A**), including classical (MNPa) and non-classical (MNPb) monocyte derived phagocytes, typically of pro-inflammatory M1 macrophage phenotype (32). Enrichment of dendritic cells (MNPc) was also seen, supporting the enrichment of the *antigen processing and presentation* pathway identified in the ORA. Tissue macrophages (MNPd) were also enriched. Whilst classically defined as M1 or M2, there is a spectrum of macrophage polarization depending on stimulus (33). To explore potential macrophage phenotypes associated with severe kidney IRI, we undertook GSEA using curated cytokine-specific macrophage polarization state signature reference datasets (33) (**Supplementary Table 7**). We found pro-inflammatory M1 phenotype macrophage signatures to be enriched in this cohort, predominantly those polarized by *TNF*, *IFNgamma* and *Prostaglandin-E2* (**Figure 2B**). In addition to MNPs, adaptive immune subsets were enriched, including CD8 and CD4 T cells, B cells, NK cells, highlighting the importance of adaptive immune cells in severe IRI and innate-adaptive cross talk, as highlighted in the earlier ORA.

### Enrichment of innate immune cell signatures during severe IRI persists at six hours of machine perfusion

Our analyses highlight the central role of the innate immune system, in particular monocyte/macrophage activation, in the pathogenesis of severe IRI and its clinical manifestation, DGF. Next, we sought to validate the pro-inflammatory pathway activation and cellular landscape observed during severe IRI in the RCT cohort. Given all kidneys suffer IRI during NMP, and in the absence of another clinical kidney NMP cohort, we analyzed an external cohort of machine perfused kidneys (initially procured for transplant but subsequently declined and offered for research; **Supplementary Table 9**). These were part of a study assessing RBC compared to acellular perfusate, the full analysis of which is beyond the scope of this study. The cohort was predominantly male (57%) with a mean age of 65 years, median BMI 24.7kg/m^2^ and a median CIT of 28 hours. First, we confirmed that kidneys perfused with RBC based versus acellular perfusates shared similar expression patterns at bulk transcriptomic analysis at both the pre-NMP and six-hour NMP timepoints. Furthermore, on PCA there was no separation of samples at the six-hour timepoint based on perfusate used (**Figure 2C**). Some separability was seen between donor gender (**Figure 2D**), and this was subsequently included as a co-variate for the differential expression model. After six hours of perfusion, only 4 DEGs were identified between RBC and acellular perfused kidneys, namely hemoglobin related genes (HBA1, HBA2, HBB; **Figure 2E**). Given the lack of significant transcriptomic difference between RBC and acellular perfused kidneys, they were considered together as one cohort for this study.

**Figure 3:**
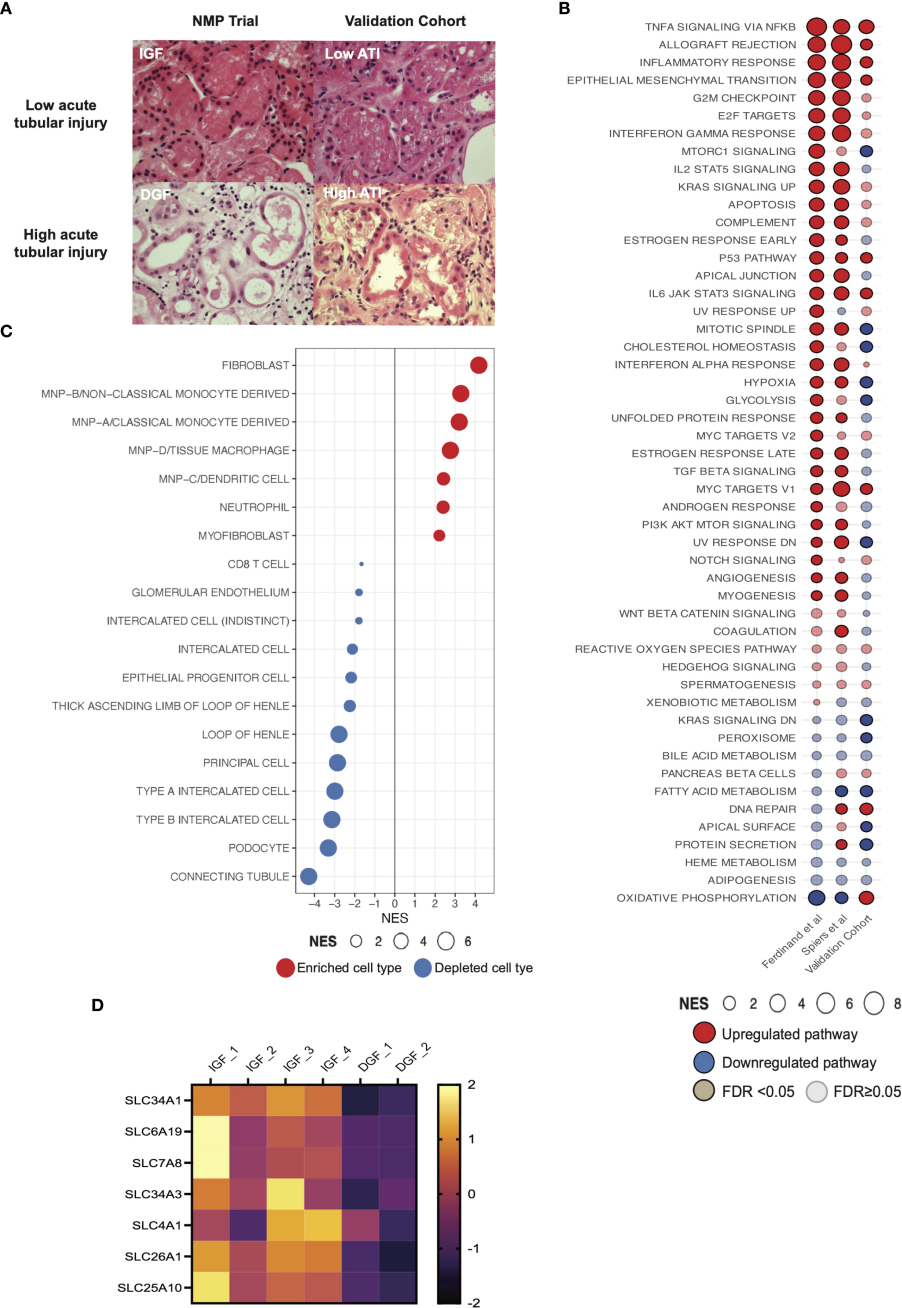
Pro-inflammatory pathway signaling and enrichment of innate immune cell signatures in an external validation cohort of machine perfused kidneys. **(A)** Histological images of kidney tissue with hematoxylin & eosin stain. Top panels show representative biopsies from kidneys with low acute tubular injury (ATI) and IGF; lower panels show representative biopsies from kidneys with high ATI and DGF. IGF, immediate graft function; DGF, delayed graft function. Magnification x40. **(B)** Comparison of GSEA against the Hallmarks datasets between kidney NMP datasets, including Ferdinand et al. (8), contemporary analysis and validation cohort (**Supplementary Tables 3, 8**). Solid color represents FDR <0.05, transparency represents FDR ≥0.05. **(C)** GSEA of differentially expressed genes in high acute tubular injury kidneys from the validation cohort against curated scRNAseq signatures (32). Only significant cell types are plotted (FDR <0.05). Size of bubble corresponds to degree of enrichment. NES, normalized enrichment score; FDR, false discovery rate. **(D)** Heatmap depicting z scored expression of solute transporter genes between graft function types.

We observed that kidneys from the RCT with mild IRI and IGF had lower levels of ATI on histological examination of biopsies taken at 1 hour of NMP (biopsies taken in tandem with biopsies used for RNAseq), whilst kidneys with severe IRI and DGF had higher severity and greater geographical distribution of ATI. Thus, the degree of ATI was used as a histological surrogate endpoint of IRI severity and graft dysfunction in the external cohort. Biopsies at 6 hours of NMP in this cohort were assessed by a blinded specialist transplant histopathologist (AP) for degree of acute tubular injury (see Histology Assessment in methods) (34). Based on this histological grading, the cohort was divided into low ATI (n=6) and high ATI (n=7) groups. Compared to donors of kidneys with low ATI, high ATI kidney donors were predominantly female DBD donors and had a trend towards older age and longer cold ischemic times; full comparison can be found in **Table 2**. Low ATI kidneys were found to share histological similarities with mild IRI IGF kidneys, and high ATI kidneys were histologically similar to severe IRI DGF kidneys from the clinical NMP trial cohort (**Figure 3A**); demographics for all kidneys included in these groups for transcriptomic analysis are presented in **Tables 1**, **2**. We performed comparative transcriptomic analysis of tissue biopsies taken at the 6 hours machine perfusion timepoint in the external cohort, and analyzed gene expression signatures between low and high ATI kidneys.

**Table 2:** Demographic comparison of research kidney donors.

Research kidney cohort		*Low ATI*	*High ATI*	*P value*
*Total n (%)*	-	6 (46.2)	7 (53.8)	-
*Donor age (yrs)*	Median (IQR)	65.5 (62.0 to 69.0)	74.0 (65.5 to 74.5)	0.130
*Gender*	Female	0 (0.0)	6 (85.7)	0.005
	Male	6 (100.0)	1 (14.3)	
*BMI (kg/m2)*	Median (IQR)	24.8 (24.6 to 26.4)	23.3 (23.1 to 26.6)	0.280
*Donor Type*	DBD	2 (33.3)	7 (100.0)	0.021
	DCD	4 (66.7)	0 (0.0)	
*Donor Cause of Death*	Hypoxic brain damage	3 (50.0)	2 (28.6)	0.790
	Intracranial thrombosis	1 (16.7)	3 (42.9)	
	Intracranial haemorrhage	2 (33.3)	2 (28.6)	
*Pre-retrieval Creatinine (mg/dl)*	Median (IQR)	65.0 (50.8 to 68.0)	57.0 (51.0 to 109.0)	0.942
*Cold Ischaemia Time (mins)*	Median (IQR)	1513.5 (938.8 to 1755.2)	1774.0 (1201.0 to 1911.0)	0.428

Table outlining comparison of demographics between acute tubular injury (ATI) groups. BMI, body mass index; DBD, donation after brain stem death; DCD, donation after circulatory death; IQR, inter-quartile range.

High ATI compared to low ATI kidneys showed enrichment of *TNFalpha signaling via NFkB, Inflammatory response* and *Allograft rejection* pathways (**Figure 3B**), recapitulating the severe IRI signature seen in DGF kidneys from our NMP RCT derivation cohort, and in a prior study from the same trial (8). Deconvolution of DEGs at 6 hours NMP in high ATI kidneys from the external cohort (severe IRI kidneys) revealed consistent enrichment for mononuclear phagocyte signatures (**Figure 3C**), similar to those observed during NMP in RCT kidneys with severe IRI and developing DGF. These findings further support the role of inflammatory innate immune cells of myeloid lineage in the pathogenesis of severe IRI, and recapitulate the pro-inflammatory transcriptomic signature observed in the clinical NMP kidney cohort. The analysis also suggests the innate immune cell signature present at one hour of machine perfusion (as seen in the RCT cohort) is sustained at six hours in severely injured kidneys (as seen in the external cohort).

### Kidneys with severe IRI show increased expression of gene signatures associated with fibroblasts/myofibroblasts and depletion of renal tubular cell signatures during NMP

Kidneys suffering more severe IRI (DGF/high ATI kidneys in respective cohorts) also showed enrichment for fibroblasts and myofibroblasts (**Figures 2A**, **3C**). The global signature of severe IRI in both the previous (8) and the current DGF cohorts, and in the high ATI cohort, included enrichment for *epithelial-mesenchymal-transition* (EMT, **Figure 2C**), shared by fibroblasts and myofibroblasts in kidney fibrosis (35).

We observed depletion of several renal tubuloepithelial cell (TECs) signatures during NMP in kidneys with severe IRI that went on to develop DGF (**Figure 2A**). This was recapitulated in the validation cohort (**Figure 3C**). Interestingly, genes downregulated during NMP in the DGF cohort included those encoding solute carrier-mediated transmembrane transport proteins (**Figure 3D**). Genes such as *SLC4A1* and *SLC7A8* are predominantly expressed in the tubuloepithelial cells, and *SLC26A1, SLC34A1 and SLC34A3* are specifically expressed in proximal convoluted tubular cells (36); both cell populations were depleted in the severe IRI DGF kidney cohort. These genes are annotated to the ‘*transport of inorganic cations/anions and amino acids/oligopeptides*’ pathway (Reactome:R-HSA-425393), encompassing acid-base balance, glucose, and protein absorption in the tubuloepithelial compartment. In agreement, ORA of significantly downregulated genes revealed depletion of this pathway in severe IRI kidneys.

## Discussion

Ischemia-reperfusion injury manifests as post-transplant graft dysfunction and, in severely injured grafts, can lead to poorer long-term outcomes. We capitalized on a unique biobank from the only clinical RCT of kidney NMP, dissecting the mechanisms of IRI during *ex situ* NMP and characterizing transcriptomic differences between mild and severe IRI, as annotated by development of IGF or DGF post-transplant. We recapitulated previously described global pro-inflammatory signatures associated with severe IRI/DGF following kidney NMP (8) and went further to suggest a core transcriptional signature of severe IRI, including *TNFalpha signaling via NFkB, Allograft rejection* and *Inflammatory response*. In conjunction with this data, over-representation analysis showed upregulation of pathways associated with innate immune system activation, innate-adaptive immune crosstalk and propagation of inflammation in severe IRI. Deconvolution of bulk RNAseq using scRNAseq signatures suggested enrichment of proinflammatory mononuclear phagocytes, fibroblasts and myofibroblasts and relative depletion of tubular epithelial cell signatures in kidneys with severe IRI and DGF. These findings were recapitulated in tissue biopsies during NMP of kidneys with high versus low ATI, histologically similar to DGF and IGF kidneys respectively, supporting further the transcriptional signature and cellular landscape underpinning severe IRI.

NMP provides a unique platform for the multiparametric assessment of kidneys pre-transplant (6). A multitude of factors predispose kidneys to and determine the severity of IRI (including donor factors, organ procurement and subsequent cold storage), but there was limited understanding of the biological processes and cellular landscape underpinning the IRI process during NMP. Mechanistic understanding of IRI during NMP is required for the development of *ex situ* delivered therapeutic interventions using the NMP platform, and quantification of IRI severity at the transcriptomic level may be used as a surrogate endpoint to assess therapeutic efficacy. The transcriptomic signature of kidney IRI during *ex situ* NMP described here may serve this purpose. The innate immune response plays a key role in kidney injury (37), with NFkB signaling mediating many cellular responses that propagate inflammation (38). In kidney IRI recapitulated during NMP, we saw strong upregulation of NFkB signaling and downstream pro-inflammatory genes (e.g. IL1B, TNFa, CXCL8/IL8), as observed in other organ systems (39, 40). This highlights NFkB signaling as a therapeutic target in solid organ transplantation. Many approaches to NFkB inhibition have been trialed (41), but systemic administration is compromised by off-target effects making translation challenging. Targeting NFkB during NMP removes the risk of systemic toxicity (42, 43), and could be enhanced by targeting key cells implicated in IRI (44).

There is currently no evidence that NMP positively impacts kidney outcomes, but rather limits exposure to static cold storage, allowing expansion of preservation time. Our data suggest that genes and pathways associated with IRI are upregulated after one hour of NMP and persist at six hours in severely injured grafts, as seen in the liver during NMP (45), suggesting a potentially common signature of severe IRI across solid organs that persists after reperfusion. We demonstrated that kidneys with severe IRI had significant enrichment for pro-inflammatory innate immune cell signatures during NMP, implicating inflammatory cells of myeloid lineage in IRI pathogenesis. Interestingly, innate immune cell signatures present at one hour of NMP were also present after six hours of NMP in severely injured kidneys (in the independent NMP cohort of high ATI kidneys), suggesting sustained activation, as seen in murine models *in vivo (* 46). This requires further investigation, including consideration of how graft quality impacts IRI, with NMP serving as a platform for organ assessment and targeted amelioration of molecular and cellular events related to IRI.

In kidneys with poor long-term function (low eGFR), which correlates with severe IRI and DGF, biopsies taken immediately post-retrieval demonstrated enrichment of tissue macrophage, NK cell and adaptive immune cell signatures (47), as observed in kidneys with more severe IRI in our study. This may suggest activation of immune cell populations starts at donor organ procurement, particularly in marginal kidneys, and is further exacerbated during reperfusion, leading to severe IRI. Importantly, kidney biopsies at the time of organ procurement in allografts with low 12-month eGFR (47), showed enrichment for fibroblast/myofibroblast and EMT transcriptomic signatures, similar to our observations in kidneys during NMP that develop DGF or those with severe ATI (validation cohort). This observation suggests the process of pro-fibrotic gene induction could start at organ procurement and, in the context of the pro-inflammatory pathway upregulation and dysregulated innate immune activation seen upon reperfusion, impaired healing may be sustained in kidneys suffering severe IRI, leading to poor long-term function.

An important limitation of this unbiased exploratory study is the small number of kidneys in the DGF cohort. This reflects the challenges with biobanking in a real-world clinical setting and the need to support multiple translational studies. We also removed one DGF sample as we wished to focus on DGF related to severe IRI rather than post-transplant contributors to DGF. To address the limitation of small sample size, in the absence of additional samples to increase the cohort, we relied on the convergence and robustness of bioinformatics analyses (22) and on internal variation assessment approaches such as the weighted likelihood empirical Bayes method. This statistical approach achieves highly stable and replicable results even for experiments with very small numbers of biological replicates (48), as reflected in the fact we were able to confirm previously detected global transcriptomic signatures associated with DGF that were derived from a larger subcohort of the same RCT (8). We were then able to replicate the transcriptomic and cell expression signatures established using this method in the external validation cohort, of non-transplanted NMP kidneys with high ATI (histologically similar to DGF kidneys). The validation cohort contained a larger number of samples, highlighting the reproducibility of transcriptomic findings obtained in the smaller derivation cohort. Our findings would have been strengthened by further validation at the protein level, e.g. to explore localization of immune cells in the tissue, and this should be included in future studies of IRI during NMP. Finally, although this study focused on IRI during NMP, we acknowledge that the IRI process at kidney implantation is more complex and influenced by recipient factors, including recipient immune cells.

In summary, we describe a core transcriptomic signature of IRI during NMP in severely injured kidneys, enriched for innate immune processes. We explored the cell expression landscape of kidneys suffering severe IRI during NMP, including cell expression signatures enriched for pro-inflammatory innate immune cells, fibroblasts/myofibroblasts and depleted for tubuloepithelial cells. Integrating this knowledge with future studies examining organs during *ex situ* NMP may facilitate development of targeted therapeutics for the improvement of organs prior to transplantation.

## Data Availability

The datasets presented in this study can be found in online repositories. The names of the repository/repositories and accession number(s) can be found below: https://www.ncbi.nlm.nih.gov/geo/, GSE293480.

## Supplementary material

Supplementary Figure 1 Kidney machine perfusion and sampling regime. **(A)** Schematic of normothermic machine perfusion of human kidneys and associated sampling regime. Bulk RNAseq represents those kidneys sent for sequencing and the final cohort those that were included in the final analysis. **(B)** Schematic of human kidney machine perfusion and sampling regime for red blood cell and acellular solution perfused kidneys. In both plots, blue arrows represent RNA extraction and quantification step, purple arrows represent submission for sequencing, green arrows represent post-sequencing technical quality control. Created in https://BioRender.com.

Supplementary Figure 2 Quality control and initial analysis of tissue bulk RNAseq data. FastQC was used for quality control of bulk mRNA sequencing outputs including sequence counts **(A)** and GC content **(B)**. MultiQC aggregation of assignment **(C)** and alignment **(D)** of reads to the *H Sapiens* reference genome. Assessment of gene expression between samples by Jaccard similarity index **(E)** and principal component analysis [PCA; **(F)**] using top 500 most variable genes. **(G)** Heatmap representation of top 200 differentially expressed genes between initial cohort of IGF and DGF samples. **(I)** PCA of final bulk mRNAseq cohort.

Supplementary Figure 3 Validation of global transcriptomic signature of severe IRI/delayed graft function in kidneys during normothermic machine perfusion. **(A)** Comparison of GSEA against the Hallmarks pathways using differentially expressed genes in DGF kidneys. Ferdinand et al. data plotted from associated supplementary data (8). Size of bubble corresponds to degree of enrichment. NES, normalized enrichment score; FDR, false discovery rate. Solid color represents FDR <0.05, transparency represents FDR ≥0.05. Full data available in **Supplementary Table 3**.
