# Supplementary figures and images for: Transcriptomic profiling during normothermic machine perfusion of human kidneys reveals a pro-inflammatory cellular landscape and gene expression signature associated with severe ischemia-reperfusion injury and delayed graft function
Source: Front Immunol. 2025 Oct 22;16:1679251. doi: 10.3389/fimmu.2025.1679251 (PMC12586191; doi:10.3389/fimmu.2025.1679251)

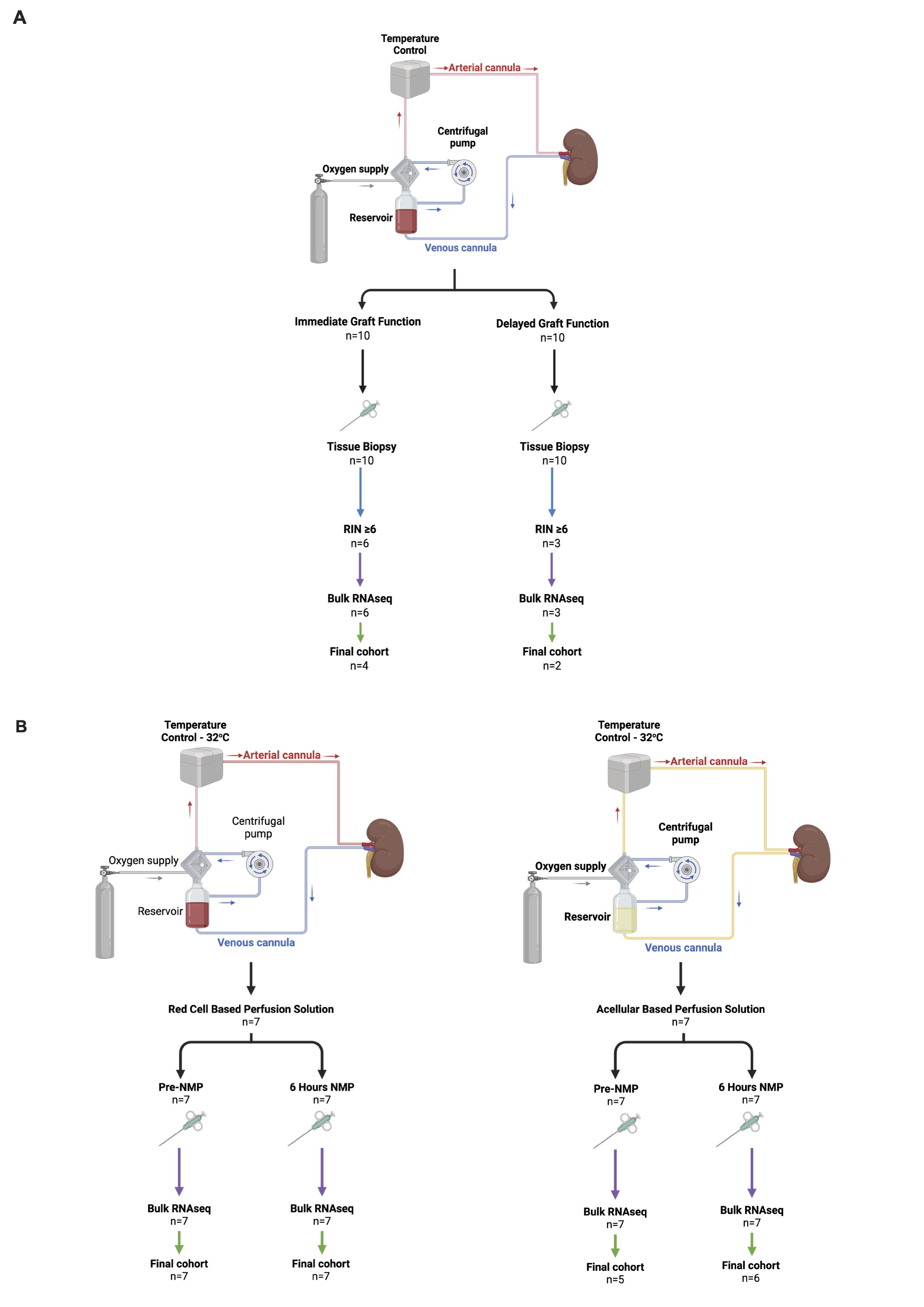

Supplement: Supplementary Figure 1 — Kidney machine perfusion and sampling regime. (A) Schematic of normothermic machine perfusion of human kidneys and associated sampling regime. Bulk RNAseq represents those kidneys sent for sequencing and the final cohort those that were included in the final analysis. (B) Schematic of human kidney machine perfusion and sampling regime for red blood cell and acellular solution perfused kidneys. In both plots, blue arrows represent RNA extraction and quantification step, purple arrows represent submission for sequencing, green arrows represent post-sequencing technical quality control. Created in https://BioRender.com. [file Image1.jpeg]

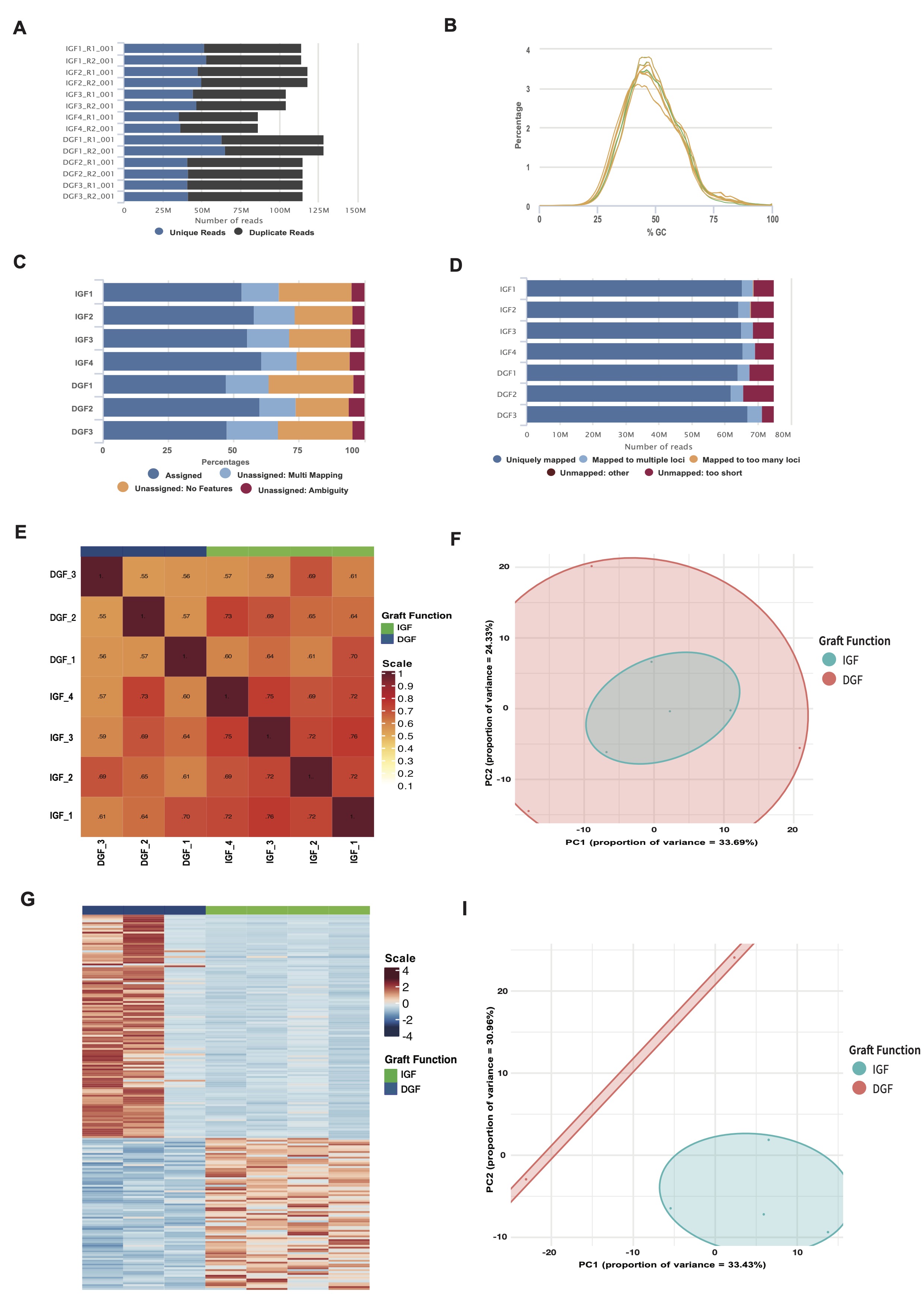

Supplement: Supplementary Figure 2 — Quality control and initial analysis of tissue bulk RNAseq data. FastQC was used for quality control of bulk mRNA sequencing outputs including sequence counts (A) and GC content (B). MultiQC aggregation of assignment (C) and alignment (D) of reads to the H Sapiens reference genome. Assessment of gene expression between samples by Jaccard similarity index (E) and principal component analysis [PCA; (F)] using top 500 most variable genes. (G) Heatmap representation of top 200 differentially expressed genes between initial cohort of IGF and DGF samples. (I) PCA of final bulk mRNAseq cohort. [file Image2.jpeg]

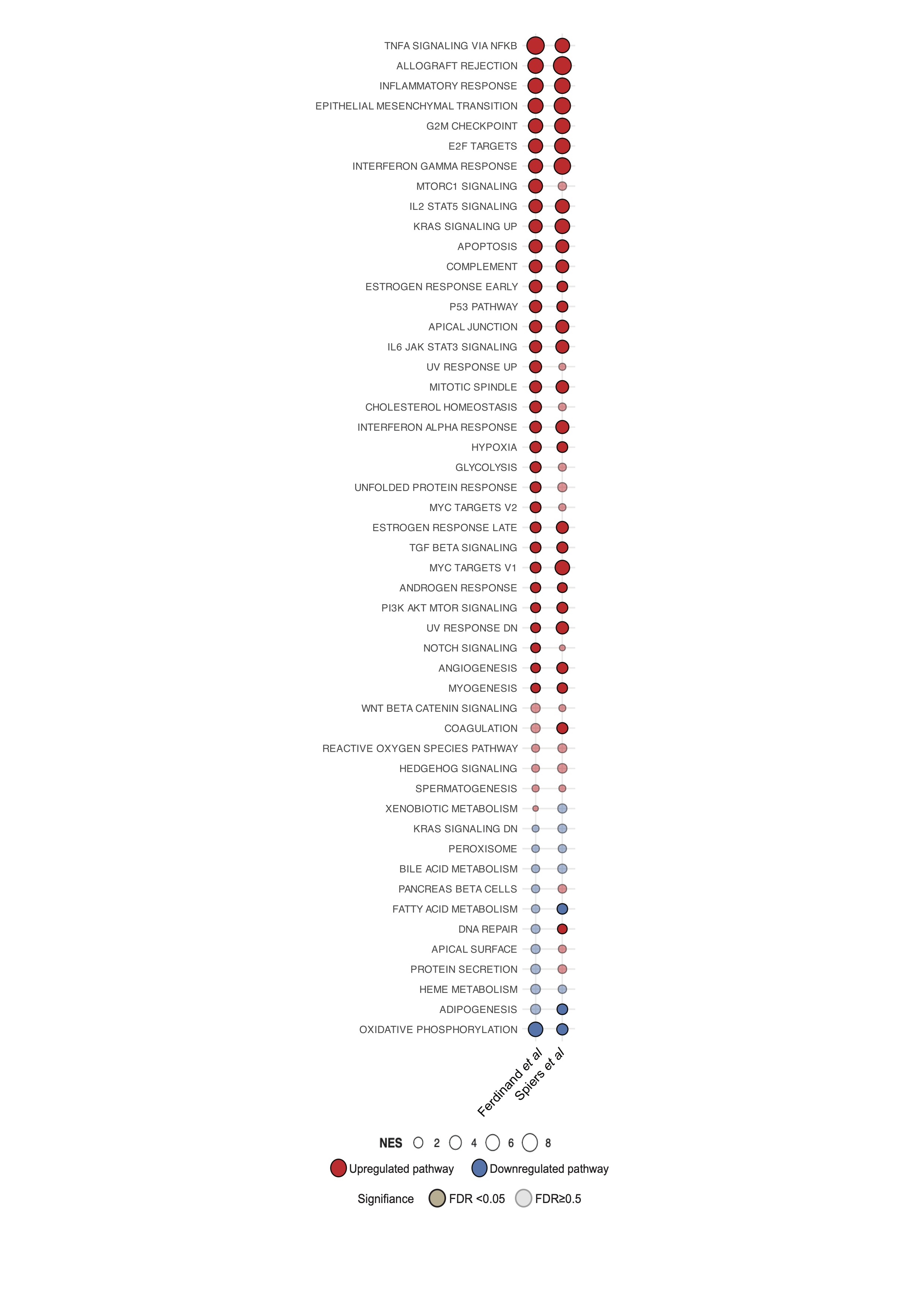

Supplement: Supplementary Figure 3 — Validation of global transcriptomic signature of severe IRI/delayed graft function in kidneys during normothermic machine perfusion. (A) Comparison of GSEA against the Hallmarks pathways using differentially expressed genes in DGF kidneys. Ferdinand et al. data plotted from associated supplementary data (8). Size of bubble corresponds to degree of enrichment. NES, normalized enrichment score; FDR, false discovery rate. Solid color represents FDR <0.05, transparency represents FDR ≥0.05. Full data available in Supplementary Table 3 . [file Image3.jpeg]
